# BMI or not to BMI? debating the value of body mass index as a measure of health in adults

**DOI:** 10.1186/s12966-025-01719-6

**Published:** 2025-02-25

**Authors:** Carmen Byker Shanks, Meg Bruening, Amy L. Yaroch

**Affiliations:** 1 Center for Nutriton & Health Impact, 14301 FNB Parkway, Suite 100, Omaha, NE 68154 USA; 2 Department of Nutritional Sciences, College of Health and Human Development, Penn State University, University Park, PA USA

**Keywords:** Body mass index, Public health intervention, Weight bias, Nutrition, Physical Activity

## Abstract

Body mass index (BMI) is used across public health to calculate height to weight ratio and translate into weight status. Whether BMI is appropriate as an individual- or population-level health measure for adults is debated. BMI is a cost-effective and feasible metric to establish health risk. Yet, BMI’s historical underpinnings, weight categories, usefulness as clinical diagnostic measure, and application across population subgroups has called the measurement tool into question. At the annual ISBNPA meeting in June 2023, the co-authors engaged in a debate session on the topic. This paper presents the complexity of arguments for or against BMI as a measurement tool and proposes its evolution to support whole-person health.

## Background

Whether body mass index (BMI) is appropriate as an individual- or population-level health measure for adults is hotly debated in the news, amongst professional organizations, as well as in the scientific community. In June 2023, the three co-authors on this paper attended the ISBNPA Conference in Uppsala, Sweden and participated in a debate session titled, “BMI or not to BMI.” This paper highlights the key points discussed during the debate, outlines paths forward for utilizing BMI in the field of behavioral nutrition and physical activity, and it is not intended to be a scoping review of the literature.

The pros for BMI? Many argue that BMI is a cost-effective and feasible metric to establish health risk. Moreover, there is potential to continue to build upon and improve BMI as a measure, as well as its implementation in public health settings. The cons? Publications and statements have called into question the utility of BMI, its weight categories, usefulness as a clinical diagnostic measure, and its inappropriate application across population subgroups. Furthermore, debates about the suitability of BMI typically provide a historical perspective which underscore the questionable foundations of the metric [1].

What is BMI? BMI is a person’s weight in kilograms divided by the square of that person’s height in meters [2]. BMI does not measure body fat but rather is a proxy for body fat by calculating a height to weight ratio. BMI strongly correlates with body fatness, although this relationship varies by age, sex, or race/ethnicity [3]. BMI calculations are translated into a number (below 18.5 to 30 and above) which correspond with weight status categories. Lower values can denote less body fatness, translating into underweight (below 18.5) or healthy weight (18.5–24.9) status interpretations. Higher values can denote more body fatness, translating into overweight (25.0-29.9) or obesity (30 or greater) weight status interpretations. Further, BMI is used as a continuous variable to examine the impact of small changes in BMI on health outcomes, and as a categorical variable to compare health outcomes across different weight categories. While using BMI as a continuous variable can provide insights into the relationship between small changes in BMI and health, interpreting the significance of these changes can be challenging. On the other hand, when BMI is used as a categorical variable, the arbitrary cut-offs may not be applicable to all populations, leading to potential misclassification or misinterpretation of health risks.

The historical underpinnings of BMI can be traced to 1823, when Adolphe Quetelet, a Belgian scientist practicing astronomy, math, and sociology, introduced the concept of the Quetelet Index, essentially a precursor to BMI [4]. While the Quetelet Index is not identical to BMI, it did lay the foundation for BMI’s future development. In his quest to determine the main characteristics of the “average man,” Quetelet did not select from a diverse sample; he generated his index drawing from the population within reach, namely Western European men. Therefore, the Quetelet Index lacked representativeness and generalizability. A few decades later, Francis Galton used the Quetelet Index to spawn and promote what is now known as eugenics [4]. His goal was to use normal distribution of a variable that characterized people broadly, divide it into quartiles, and then rank and compare the variable. Fast forward to the 1970’s, when Ancel Keys, an American physiologist and nutritionist, proposed a simplified version of the Quetelet Index [5–7]. This new measure became known as BMI and from the start, it gained widespread popularity, momentum, and use.

Today, BMI is applied across public health worldwide, even though complementary measures exist including waist-to -hip and -height ratios, body fat percentage, dual-energy X-ray absorptiometry, skinfold, bioelectrical impedance analysis. The global use of BMI has made it clear that excess body fat, as indicated by higher BMI categories, is a significant contributor to health risks such as cardiovascular disease and diabetes. At the population level, rates of people with obesity, as measured by BMI, have continued to rise globally over the last several decades [8]. To address the obesity epidemic, diet and/or physical activity interventions have been implemented among a variety of populations across the prevention and treatment spectrum. Many of these same interventions have used BMI as their objective “gold standard” measure of obesity. International organizations, such as the World Health Organization, have used BMI as a global health indicator [9]. Funding agencies and public health organizations have buoyed BMI measurement in funding announcements, white papers, and assessment tool recommendations. Primary care clinics post BMI charts on the wall and doctors have discussions about BMI with patients. In-home scales can calculate BMI to help to promote behavior change in nutrition and physical activity.

BMI’s widespread application has perpetuated its use, but its appropriateness is debated. This debate is not binary and there is rationale on both sides. The spectrum of the debate is presented so that we can be more intentional and impactful in our work to improve nutrition and physical activity behaviors and ultimately, population-level health and well-being.

## Debate: BMI or not to BMI?

### Point: why does BMI remain as the status quo?

BMI can help establish risk. What evidence exists about how the index contributes to BMI-related health risk? Several risk factors have been drawn from associations with the obesity category as calculated from BMI [10]. It is well established that obesity puts individuals and populations at risk for many serious chronic diseases and illnesses. Obesity is a risk factor for type 2 diabetes, cardiometabolic outcomes, and cancers [11–13]. Further, taking COVID-19 as an example, obesity increases the risk of severe illness from COVID-19 [14]. Having obesity triples the risk of COVID-19 hospitalization. Lastly, BMI has been predictive of future morbidity and death [15]. Although BMI is not a diagnostic tool, it has been used to establish urgent risks programming for population health.

BMI is a cost-effective and feasible measurement tool. It is a simple and inexpensive proxy measure of body fat [16]. BMI relies solely on height and weight. To calculate both, access to the proper equipment (i.e., scale and a stadiometer) is required, and both are relatively inexpensive. Individuals can have their BMI routinely measured and calculated with reasonable accuracy by individuals with little training. In large part because of these reasons, the widespread and longstanding, cost-effective application of BMI contributes to its utility for tracking at the population level. In the United States of America (USA), the Centers for Disease Control and Prevention (CDC) maps are extensively used images that show changes in overweight and obesity rates annually and over time [17]. Worldwide, BMI is tracked graphically to understand aspects of population health. BMI’s use has resulted in an increased availability of published population data that allows public health professionals to make comparisons across time, regions, and population subgroups at low cost.

### Counterpoint: BMI measurement is flawed.

BMI relies on height and weight to measure individuals, but it does not consider lean mass, bone, fat proportions, nor metabolic health—strong indicators for overall health [18, 19]. BMI’s weight categories imply that there is a typical healthy, body, a concept that oversimplifies health by disregarding the variety of human body types. Using BMI as a primary indicator for health has the potential to reinforce harmful societal norms about the “ideal” body. Related, BMI’s ties to the eugenics movement should cause pause. While BMI was not created for use in eugenics, its developmental history reflects eugenic ideology, particularly in how it categorizes body size and shape into an “ideal” body type.

Examining BMI by category is not precise to change as it relies on broad classifications. Examining BMI as a continuous measure provides more nuanced information about changes in the weight to heigh ratio. Still, BMI in both instances does not account for body composition or distribution of body fat, which also impacts health.

Reviews demonstrate intervention efficacy for nutrition and physical activity behaviors and other more sensitive biometric outcomes, but most studies show limited improvement in BMI, if any [20, 21]. This results in confusion about effective strategies to improve health outcomes when BMI is the primary measure. The Health at Every Size literature has well-documented the need and benefit for approaches that emphasize health-promoting behaviors versus weight-based interventions across various human body types [22].

The lay public and some individual allied health professionals may lack sensitivity when interpreting BMI or misunderstand the limitations of its application [23, 24]. Misguided interpretations of BMI’s weight categories may be used as an indicator of overall health. Overinterpretation may lead to perpetuated weight stigma, or discriminatory acts or perceptions based upon weight and size. To address these issues, other cost-effective and non-invasive measures that shift the conversation to more holistically portraying associations between body composition and health are useful. For instance, waist circumference—while having its own strengths and limitations (such as being relatively inexpensive, sensitive to health outcomes and changes in body composition, but prone to inconsistencies in measurement)—provides a more accurate reflection of fat distribution and could complement BMI when used together [25]. Another alternative measure gaining recent attention is the body roundness index [26]. Developed about a decade ago, it is a non-invasive anthropometric measure which is based on waist and potentially hip circumference, along with height (but not weight). These measurements are taken on individuals and then put into a formula to estimate visceral adipose tissue, which is associated with various chronic diseases. In fact, a recent national cohort study using self-reported data from the National Health and Nutrition Examination Survey (NHANES) among approximately 33,000 USA adults over 20 years found a U-shaped association with the body roundness index and all all-cause mortality risk [27]. Both measures do not require the measurement of weight at all, with can help address weight stigma associated with BMI.

Due to BMI’s tumulted developmental history, the applicability across diverse racial, ethnic, and gender groups is debated. Furthermore, the foundation of BMI weight categories is precarious, as curves and cut-offs are standardized to White European, mainly male populations, limiting the generalizability of BMI as a tool to various populations [6]. Admittedly, much of modern medicine has been grounded in research primarily conducted on Western European men, leading to a narrow perspective on health that may not be generalizable to other racial, ethnic, and gender groups. Thus, BMI as a measure of health may perpetuate inequities among populations that are already underrepresented in medical and behavioral research [28].

There are some populations groups in which inconsistent relationships exist between BMI and health outcomes. For example, adverse health outcomes are associated with BMIs in the “under” or “normal” weight BMI category for populations of Asian descent [29]. For Hispanic populations, one study found mortality risk has a mixed BMI threshold across studies and waste to hip ratio is a better predictor of risk [30]. Since women tend to differ in muscle mass and body fat, BMI and its association with health risks may be less accurate for women [3]. Although findings about racial, ethnic, and sex differences are not completely consistent across studies, BMI research points to the value of additional measures—waist circumference, waist-to-hip-ratio, body fat percentage, metabolic health—in understanding body composition and health risk [31–33].

### Joint conclusion: evolution of BMI to support whole-person health

Given the complexities surrounding BMI, the authors argue for reconsidering BMI as a standard measure to establish health risk. It is more palatable to support application of BMI if we discuss how the status quo use of the measure needs to change and how it might evolve. Here, challenges, assets, and changes needed for BMI to continue to be a feasible and relevant measure are explored.

Evolution and improvement of the BMI measures is warranted. Recently, the World Health Organization adopted a tailored body mass index for Asian populations where the range for overweight begins at 23 and the range for obesity begins at 25 [34]. This tailored range exists because of the established evidence around the relationship between diabetes development and BMI in Asian population subgroups. Other research has begun to determine appropriate tailored cutoffs for diverse racial and ethnic groups.

BMI can evolve by taking other measures of fat and skeletal muscle into account alongside BMI in the clinical setting. A retrospective cohort study investigated BMI, ponderal index, visceral fat area, subcutaneous fat area, and liver volume as potential predictors of obesity-related comorbidities. The study concluded that more targeted measures are necessary to accurately predict these comorbidities [35]. Recently, the American Medical Association (AMA) adopted a new policy that supports the approach of incorporating other measures of fat and skeletal muscle alongside BMI to determine health risks [36]. This policy supports that BMI, especially its classifications, has induced historical harm with racist underpinnings, should be used only with other measures of adiposity (e.g., waist circumference, body composition), and should not be used as a sole measure for insurance reimbursement. This position is a significant shift in USA approaches for using BMI and will take time to implement in health-care settings.

The BMI classification can evolve by using other measures alongside it that account for the structural and social determinants of health. There is more to health than weight and height, such as where and how people live, work, and play, along with how they access services such as healthcare. Breslow (2006) argues that in the third era of health, which focuses on health as a resource to improve our capacity to live, measurement, including BMI, should evolve and include a set of indicators rather than a single index of health, and more recent research has reflected the same sentiment [37, 38]. In practice, BMI can be one way to understand health but should be considered with other factors (e.g., physical activity, diet, sleep) and complementary measures (e.g., fat and fat-free mass through methods such as dual-energy X-ray absorptiometry, skinfold, bioelectrical impedance analysis) to determine health risks.

To move forward with BMI as a complementary measure for health, improvements in implementation are required. Weight stigma refers to the discriminatory acts targeted towards individuals due to their weight and size and is experienced by over 40% of adults in the US [39, 40]. This is not the current intent of BMI but can be an unfortunate side effect if the results are delivered in a way that is judgmental or reinforces negative societal norms about body size. As BMI has the potential to invoke weight stigma, it is important for researchers and practitioners to counteract this deleterious effect. For scientists, one important way to understand a construct such as weight stigma is to measure it. For instance, the Gus Schumacher Nutrition Incentive Program (GusNIP) in the USA developed a weight stigma toolkit, which compiles a variety of measures that are available to understand weight stigma [41]. Resources in this toolkit can be used to elucidate weight stigma alongside BMI. Following, it is important to develop strategies to mitigate weight stigma, particularly for populations vulnerable for misclassification of health by the BMI measure. For instance, complementing BMI categories with other measures that offer a broader picture of health may shift the focus away from stigma associated with weight.

Audience appropriate resources at every level of the socioecological model should be used alongside BMI [42]. At the individual level, weight stigma can be reduced by using first person language when discussing BMI. At the interpersonal level, families, friends, and communities can check the assumptions made about people at every size in communication with each other. At the institutional level, public health professionals need training and language to focus on health behaviors rather than weight. Addressing health needs to be people-centered, empathetic, motivate behavior change, and not perpetuate weight stigma—focusing solely on BMI may not accomplish these objectives. At the societal level, the media can change the way that weight is discussed. At the policy level, policies need to be strengthened to support weight inclusion [42].

## Conclusions

The authors argue that the use of BMI could and should advance. The public health and behavioral nutrition and physical activity fields should rethink about how we can reassess, reimagine, and revise BMI. If we continue to use BMI as a primary tool of assessing intervention effectiveness and efficacy, we need to adapt it to improve its prevision and applicability with health outcomes related to diverse population groups. As scholars who aim to affect health at population-levels through improved nutrition and physical activity behaviors, our assessment of success can include BMI, but other measures need to be selected and/or developed to offer a more holistic view of health. Solely depending upon BMI has the potential to misrepresent individuals and other measures should be used alongside to provide a more comprehensive picture of health. If BMI does not evolve, further conversations and actions towards de-implementation should be considered to protect individual and population health.

## Data Availability

Not applicable.

## Abbreviations

BMI: Body Mass Index
GusNIP: Gus Schumacher Nutrition Incentive Program
USA: United States of America
AMA: American Medical Association
